# De Novo Cancer Incidence after Cholecystectomy in Korean Population

**DOI:** 10.3390/jcm10071445

**Published:** 2021-04-01

**Authors:** Yun Kyung Jung, Junghyun Yoon, Kyeong Geun Lee, Han Joon Kim, Boyoung Park, Dongho Choi

**Affiliations:** 1 Department of Surgery, College of Medicine, Hanyang University, Seoul 04763, Korea; YunKyung.Jung@mountsinai.org (Y.K.J.); hepafel@hanyang.ac.kr (K.G.L.); thicknyh@hanyang.ac.kr (H.J.K.); 2 Department of Public Health Sciences, Hanyang University, Seoul 04763, Korea; cumyluceat@hanyang.ac.kr; 3 Department of Preventive Medicine, College of Medicine, Hanyang University, Seoul 04763, Korea

**Keywords:** cholecystectomy, cancer incidence, standardised incidence ratio

## Abstract

**Background:** Cancer development after cholecystectomy remains debatable. We estimated the major cancer incidence rates after cholecystectomy stratified by age and sex. **Methods:** The records of 408,769 subjects aged >20 years were extracted from the National Health Insurance database from 2008 to 2016. The risks of major cancers were compared between the cholecystectomy and general populations using standardised incidence ratios (SIR). **Results:** The overall cancer incidence was comparable between cholecystectomy patients and the general population. However, patients aged <65 years who underwent cholecystectomy had a higher cancer risk than those aged ≥65 years and the general population (SIR 2.62; 95% confidence interval [CI] 2.15–3.08; SIR 1.36, 95% CI 1.32–1.40; and SIR 0.90, 95% CI 0.87–0.92 in men and SIR 1.91; 95% CI 1.71–2.10; SIR 1.07; 95% CI 1.03–1.10; and SIR 0.90; 95% CI 0.87–0.94 in women aged 20–34, 35–64, and ≥65 years at cholecystectomy). Colorectal and liver cancer incidences after cholecystectomy were higher than those in the general population regardless of age group and sex (SIR, 1.55 for colorectal cancer in men and women; SIR, 1.25 and 1.51 for liver cancer in men and women, respectively). However, for other major cancers, the risk was higher in patients who underwent cholecystectomy at a younger age than in those who underwent cholecystectomy at an age ≥65 years. **Conclusion:** Patients with cholecystectomy, especially those undergoing cholecystectomy at a younger age, need preventive strategies based on the cancer type.

## 1. Introduction

Cholecystectomy is the sixth most common operation in Korea and the number of operations performed per year has been increasing rapidly. The two most common causes for cholecystectomy are cholelithiasis (gallstones) and cholecystitis (inflammation of the gallbladder) [1]. This steep increment in the number of surgeries is attributed to factors such aging and changes in eating habits [2], and the number of cholecystectomies is expected to increase further. Although cholecystectomy is a common and simple operation, concerns about postoperative long-term health effects in patients undergoing cholecystectomy have been raised.

It has been suggested that cancer is one of the first health problems that occur after cholecystectomy, based on two hypotheses: first, the change in bile acid flow after cholecystectomy and the subsequent increase in duodeno-gastric bile reflux followed by direct contact of bile acid (without dilution with food) with the intestinal wall [3,4,5] may cause cancer; and second, cancer risk related to chronic inflammation (one of the common causes of cholecystectomy) [6,7]. Several studies have reported the association between various types of cancer and cholecystectomy. Specifically, these cancers included gallbladder, liver, and biliary tract cancers [8,9,10,11,12], digestive tract cancer [11,13,14,15,16,17,18,19], pancreatic cancer [20,21], prostate cancer [22], and breast cancer [23]. Risks for several cancer types, including pancreatic cancer [20,21], colorectal cancer [19], and liver cancer [24], have been reported to be associated with cholecystectomy via meta-analyses; however, available information concerning the risks for other cancer types is scarce; thus, no clear conclusions have been reached.

Recently, cancer incidence after cholecystectomy has been investigated using population-based databases, such as insurance claims databases [10,25,26], allowing investigators to determine the incidence of various types of cancer after cholecystectomy. However, previous studies using population-based databases focused on the cancer risk after cholecystitis based on the inflammation-associated cancer risk theory; however, the risk of cancer after cholecystectomy has not been well investigated. Considering that comprehensive health management is one of the important aspects for achieving long-term survival, the whole spectrum of cancer risks needs to be considered [27] in people with cholecystectomy. However, the risks of various types of cancer, especially rare cancer types, would be difficult to estimate with the data of a limited number of patients who underwent cholecystectomy.

Korea is mainly composed of a single-race population and has a single mandatory nationwide health insurance system covering about 97% of the population. Thus, using the health insurance database, the data of almost all cases of cholecystectomy and related health problems, including rare diseases, can be accessed. Therefore, this study aimed to estimate the incidence rates of all cancer types in patients who underwent cholecystectomy and to identify whether they were at a higher risk of developing any cancer type than the general population.

## 2. Materials and Methods

This was a retrospective cohort study using data from the National Health Insurance Service (NHIS) database. Since it is a compulsory requirement for all citizens, thus most of the Korean population, to subscribe to Korea’s national health insurance, the out-of-pocket was less than 20% of the total medical cost, minimizing the burden of medical services use. In addition, the NHIS database collects of all medical usage of Korean population since 2002 [28]. The NHIS database provides the data of national health screening, health care utilisation, and long-term care insurance (including qualifications and premiums, history of hospital use, national health examinations, and medical benefit) from birth to death [28]. In addition, personal characteristics of the insured population, such as age, sex, income level, place of residence, and loss of insurance eligibility are recorded.

**Figure 1:**
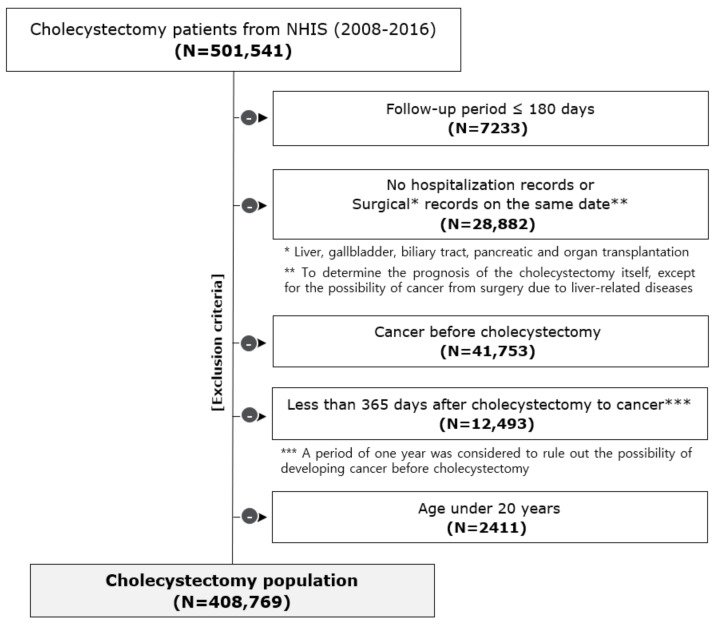
Flow diagram of the selection process of the study population. This study included 501,541 patients who underwent cholecystectomy from 2008 to 2016 using the National Health Insurance Service (NHIS) database. According to the exclusion criteria of this study, a total of 408,768 patients were included in the final study.

Figure 1 shows the flow diagram of the selection process of the study population. The data of a total of 501,541 patients with the cholecystectomy surgical code (Q7380) were extracted from January 2008 to December 2016. Individuals with follow-up periods of less than 180 days (*n* = 7233), without a hospitalisation record (*n* = 7), with records of other surgeries involving the liver, gallbladder, biliary tract, or pancreas on the same date of the cholecystectomy (to exclude the possibility of cholecystectomy due to diseases of nearby organs) (*n* = 28,875), with any cancer-related medical records before cholecystectomy (*n* = 41,753), with cancer incidence less than 1 year after cholecystectomy (*n* = 12,493) (to rule out the possibility of prevalent cancer before cholecystectomy), and those aged under 20 years (*n* = 2411) were excluded. This study was approved by the Institutional Review Board of Hanyang University, Korea (IRB No. HYI-18-110-2). Informed consent from the participants was not obtained as we analysed anonymous secondary data provided by the NHIS.

Cancer incidence was defined as a combination of disease code according to the International Classification of Disease 10th revision (ICD-10) (C00-C96) and catastrophic illness code. First, the crude incidence rate per 100,000 person-years was calculated by cancer type and age groups. The incidence of cancer in patients with cholecystectomy was compared with that in the Korean general population (data provided by the Korea Central Cancer Registry) using standardised incidence ratios (SIR) and 95% confidence interval (CI). The SIR was calculated by dividing the observed number of cases by the expected number of cases, stratified by sex. The expected number of incident cancer was calculated by applying the age-specific cancer incidence rate of the general population to each age group of cholecystectomy patients. In addition, the SIRs of the five most common cancers in each age group were presented according to age group (20–34, 35–64, and ≥65 years). SAS version 9.4 (SAS Institute, Cary, NC, USA) was used for statistical analyses.

## 3. Results

Baseline demographic characteristics of the 408,769 cancer-free patients who underwent cholecystectomy during the years of 2008–2016 are shown in Table 1. Of these, 214,842 were women (52.4%). The mean age at cholecystectomy was 53.8 years in men and 52.8 years in women. The most common age group was 40–50, which accounted for about 43.7% of the total patient population. The total follow-up duration was 5482.0 years, and the median follow-up time was 4.7 years.

### 3.1. Site-Specific Cancer Incidence after Cholecystectomy

Of the 408,769 cancer-free patients who underwent cholecystectomy, 15,055 patients developed cancer (Table 2). The crude cancer incidence rate was 902.0 per 100,000 person-years in men and 619.0 per 100,000 person-years in women. Stomach (*n* = 1494), lung (*n* = 1174), liver (*n* = 1105), colorectal (*n* = 1077), and prostate (*n* = 912) cancers were the five most common cancer types in men, with crude incidence rates of 158.9, 124.8, 117.5, 114.5, and 96.97 per 100,000 person-years, respectively; thyroid (*n* = 1498), breast (*n* = 1127), colorectal (*n* = 696), stomach (*n* = 560), and female genital (*n* = 467) cancers were the five most common cancer types in women, with crude rates of 141.1, 106.1, 65.6, 52.7, and 43.98 per 100,00 person-years, respectively.

On comparing cancer incidence between cholecystectomy patients and the general population, the male and female groups (standard incidence ratio [SIR] 1.01; 95% confidence intervals [CI] 0.99–1.03 and SIR 1.00; 95% CI 0.98–1.03, respectively) showed no significant difference in the total cancer incidence rate from the general population. The male group showed higher incidences of colorectal cancer (SIR 1.55; 95% CI 1.45–1.64), liver cancer (SIR 1.25; 95% CI 1.18–1.32), biliary tract cancer (SIR 1.22; 95% CI 1.08–1.36), melanoma and skin cancer (SIR 1.33; 95% CI 1.15–1.5), prostate cancer (SIR 1.14; 95% CI 1.06–1.21), urinary tract cancer (SIR 1.11; 95% CI 1.02–1.20), eye, brain, and central nervous system (CNS) cancers (SIR 1.37; 95% CI 1.07–1.68), and thyroid cancer (SIR 1.33; 95% CI 1.21–1.45) than the general population. Conversely, the incidences of lip, oral cavity, and pharyngeal cancers (SIR 0.83; 95% CI 0.69–0.97), stomach cancer (SIR 0.95; 95% CI 0.90–1.00), lung cancer (SIR 0.88; SIR 0.83–0.93) and lymphoid and haematopoietic cancers (SIR 0.86; 95% CI 0.77–0.95) were lower in male patients who underwent cholecystectomy than in the general population. The female group showed higher incidences of colorectal cancer (SIR 1.55; 95% CI 1.44–1.67), liver cancer (SIR 1.51; 95% CI 1.37–1.65), other digestive organ cancers (SIR 1.21; 95% CI 1.07–1.36), melanoma and skin cancer (SIR 1.40; 95% CI 1.23–1.58), mesothelial and soft tissue cancers (SIR 1.77; 95% CI 1.28–2.26), breast cancer (SIR 1.12; 95% 1.06–1.19), eye, brain, and CNS cancers (SIR 1.83; 95% CI 1.46–2.21), and lymphoid and haematopoietic cancers (SIR 1.91; 95% CI 1.68–2.13) than the general population. Conversely, the incidences of stomach cancer (SIR 0.83; 95% CI 0.76–0.90), lung cancer (SIR 0.89; 95% CI 0.80–0.97), female genital cancers (SIR 0.90; 95% CI 0.82–0.98), and thyroid cancer (SIR 0.95; 95% CI 0.90–1.00) were lower in female patients who underwent cholecystectomy than in the general population.

### 3.2. Incidences of Major Cancers after Cholecystectomy in the Male Population

Table 3 shows the SIRs of all and the five most common cancers according to age in male cholecystectomy patients and healthy subjects. Among those who underwent cholecystectomy, the most common cancer was thyroid cancer in those aged 20–34 years, stomach cancer in those aged 35–64 years, and lung cancer in those aged ≥65 years. In terms of cancer risk, patients aged <65 years who underwent cholecystectomy had significantly higher incidence rates for all cancer types than the general population (SIR 2.62, 95% CI 2.15–3.08 in the 20–34 years age group; SIR 1.36; 95% CI 1.32–1.40 in the 35–64 years age group). In contrast, patients who underwent cholecystectomy at an age ≥65 years had lower incidence rates for all cancers than the general population (SIR 0.90; 95% CI 0.87–0.92). The incidence rates of thyroid and colorectal cancers in the 20–34 years age group, those of stomach, lymphoid and haematopoietic, liver, colorectal, lung, and thyroid cancers in the 35–64 years age group, and those of colorectal and liver cancer in the ≥65 years age group were significantly higher than those in the corresponding age groups in the general population. Although the SIR decreased with increasing age, the increment pattern of colorectal and liver cancer incidence was prominent in all age groups (SIR 5.82; 95% CI 2.01–9.62 in the 20–34 years age group; SIR 2.16; 95% CI 1.97–2.35 in the 35–64 years age group; SIR 1.38; 95% CI 1.27–1.49 in the ≥65 years age group). Lung and stomach cancer incidences in the ≥65 years age group were lower than those in the general population.

### 3.3. Incidences of Major Cancers after Cholecystectomy in the Female Population

Table 4 shows the SIRs of all and the five most common cancers according to age in female cholecystectomy patients and the general population. Compared to the corresponding age groups in the general population, female patients who underwent cholecystectomy at an age <65 years showed higher incidence rates for all cancer types, whereas those who underwent cholecystectomy at an age ≥65 years showed lower incidence rates (SIR 1.91; 95% CI 1.71–2.10 in the 20–34 years age group; SIR 1.07; 95% CI 1.03–1.10 in the 35–64 years group; SIR 0.90; 95% CI 0.87–0.94 in the ≥65 years age group). The incidence rates of thyroid, breast, female genital, lymphoid and haematopoietic, and colorectal cancers in the 20–34 years age group, those of breast and colorectal cancers in the 35–64 years age group, and those of colorectal and liver cancers in the ≥65 years age group were higher among female patients who underwent cholecystectomy than in the general female population. In all age groups, colorectal cancer incidence was significantly higher in patients who underwent cholecystectomy than in the general population (SIR 5.71; 95% CI 2.61–8.81 in the 20–34 years age group; SIR 1.88; 95% CI 1.66–2.10 in the 35–64 years age group; SIR 1.40; 95% CI 1.27–1.54 in the ≥65 years age group). Stomach and lung cancer incidences were lower in women aged ≥65 years who underwent cholecystectomy than in the corresponding age group of the general population.

## 4. Discussion

The overall cancer incidence after cholecystectomy was comparable to that in the general population, while the incidences of colorectal and liver cancers were higher, regardless of age group or sex, in the cholecystectomy group than in the general population in Korea. Male and female patients who underwent cholecystectomy at an age <65 years had higher cancer incidence rates than the general population; however, those who underwent cholecystectomy at an age ≥65 years had lower incidence rates. In patients who underwent cholecystectomy at a younger age, the increase in cancer incidence compared with that in the general population was more prominent. Compared with the general population, male patients who underwent cholecystectomy at an age of 35–64 years showed higher incidences of stomach and lung cancer, whereas those who underwent cholecystectomy at an age ≥65 years showed lower incidences. In females aged ≥65 years at cholecystectomy, the SIRs of stomach and lung cancers were less than 1, suggesting a lower incidence compared with that in the general population.

Cholecystectomy can ameliorate inflammation of the gallbladder due to cholelithiasis or cholecystitis. However, increased exposure of the digestive tract to bile [3,4,5], changes in metabolic hormone levels [29], or long-lasting inflammation before cholecystectomy are possible biological mechanisms underlying increased rate of various types of cancer. However, in this study, the overall cancer risk after cholecystectomy was not increased compared with that in the general population. Previous population-based studies have shown an increase in the overall cancer risk in patients with cholelithiasis or cholecystitis [25]. In cholelithiasis patients, undergoing cholecystectomy was associated with an increase in the overall cancer risk [26]. However, these studies did not consider possible inclusion of prevalent cancer cases or the induction time to cancer development after cholecystectomy. In studies that excluded cancer cases within the 1–2 years after cholecystectomy to ensure incident cancer evaluation and to avoid the selection of cholecystectomy cases due to illness from incipient cancer, cholecystectomy was not associated with an increased overall risk of cancer [17,30].

According to the results of our study, colorectal cancer is more likely to occur in patients who underwent cholecystectomy at all ages regardless of sex, which is comparable with the results of previous meta-analyses [19,24]. In Korea, liver cancer incidence is much higher in men, and the incidence rate of liver cancer was shown to increase with increasing age [31]. Thus, liver cancer could be included among the top five cancers in men of all age groups and in women aged ≥65 years at cholecystectomy. However, in all age groups of male and female subjects, the SIR of liver cancer was greater than 1, suggesting higher risk than that in the general population (data not shown for women aged <65 years), consistent with that shown in a previous meta-analysis [12]. In this study, pancreatic cancer was included as one of the “other digestive organ cancers” due to its relatively low incidence in the Korea population [31]. When we analysed the SIR of pancreatic cancer separately from those of other digestive organ cancers, the SIR was greater than 1 regardless of sex and age, suggesting higher incidence, as shown in previous studies [20,21].

In this study, we identified a slightly increased risk of biliary tract cancer only in the male population; this is inconsistent with previous studies, which showed a much increased biliary tract cancer risk in cholecystectomy patients [8,10]. Considering that the risk of biliary cancer was prominent within 1 year after cholecystectomy and less prominent after [10], excluding incident cancer cases <365 days after cholecystectomy would be the cause of no or slight increase in risk. A study with exclusion criteria similar to those used in this study also did not find an association between cholecystectomy and the risk of cholangiocarcinoma [17]. Based on the results of the study by Chen et al. [26], it could be suggested that the incidence rates of gallbladder and biliary tract cancers were increased due to chronic inflammation, rather than due to cholecystectomy itself.

It is also important to note that the overall incidences of gastric and lung cancers were lower in both men and women in this study. Previous studies have shown that the incidence of stomach cancer after cholecystectomy was high [15] and that of lung cancer was not significantly different from that in the general population [25,26]. When these two cancer types were analysed by age group, both men and women aged <65 at cholecystectomy showed significantly higher or similar risk than that in the general population, but those aged ≥65 years showed a significantly lower risk. Similarly, the risk of female genital cancer was lower in cholecystectomy patients than in the general population, but those aged 20–34 years at cholecystectomy showed a higher SIR. Higher risk of major cancers in those undergoing cholecystectomy could be associated with severe infection in the early adult phase, especially in terms of gastric and female genital cancers [32]. Thus, more careful observation of patients undergoing cholecystectomy is needed for efficient long-term care; further studies on this population are also needed.

Few studies have investigated the association between cholecystectomy and other common types of cancer such as prostate cancer and breast cancer [22,23]. Our results showed that the patients who underwent cholecystectomy had higher risks of breast cancer, soft tissue cancer (female), prostate cancer, urinary tract cancer, thyroid cancer (male), melanoma and skin cancer, malignancies of the eye, brain and CNS than the general population. In combination with SIR by age group, overdiagnosis due to increased surveillance should be considered. A study reported that breast cancer screening was closely associated with participation in thyroid cancer screening, one of the representatives of overdiagnosis in women [33]. Thus, if overdiagnosis would be higher in people undergoing cholecystectomy, it could be expected that the SIR of both thyroid cancer and breast cancer will be higher than that of the general population. However, considering the opposite directions of the SIR of breast cancer and that of thyroid cancer in women undergoing cholecystectomy, we could expect that the effect of overdiagnosis on the SIR would be minimal.

This study was a nationwide population-based study that had clear advantages, in that it included a large population from a single nation. To the best of our knowledge, this is the first nationwide study to consider all types of cancer after cholecystectomy with the largest study population. In addition, by including all relevant populations using a population-based database which covered more than 97% of the population, selection bias could be excluded. However, several limitations of this study need to be considered. First, this study using large-scale claims data did not include other factors associated with cancer development, such as smoking, drinking, and other clinical variables for all cases. This study did not identify a causal relationship between cholecystectomy and cancer. Rather, this study was a descriptive study that investigated whether the cancer incidence would be increased in people undergoing cholecystectomy and which type of cancer would be increased. The SIR is a statistical method commonly used in cancer epidemiology to compare incidences between populations, especially when observing cancer incidence in people with certain characteristics, such as people with specific occupations, as compared to other populations. Secondly, factors leading to the requirement for cholecystectomy, such as inflammation, polyp diagnosis, and other surgical injuries, were not considered; these factors might have been associated with cancer incidence. Third, patients who underwent cholecystectomy were identified based on medical insurance codes, and cancer incidence was identified by the combination of ICD-10 codes and the catastrophic illness registry in the NHIS database. It may cause potential misclassification. However, the cost for cholecystectomy is reimbursed by the NHIS, and the number of cholecystectomies per year was comparable to that reported in the national statistics, based on the Health Insurance Review and Assessment database. The catastrophic illness registry for major diseases is related to the reimbursement of co-payment for the diagnosis and treatment of the disease; thus, relevant clinical information is needed for approval by the national insurance administration. Therefore, the operational definitions for patients undergoing cholecystectomy and cancer incidence would be valid. Fourth, to reduce the possibility of including patients who died of unidentified cancer or in whom cancer was identified during the procedure, this study excluded patients with a follow-up period of less than 180 days and those who developed cancer within a year from the date of cholecystectomy. However, this may have underestimated the cancer risk after cholecystectomy compared with that in the general population.

In conclusion, we analysed the incidence of cancer after cholecystectomy according to age and sex. Through this study, it was possible to identify that cancer occurred frequently after cholecystectomy, and the risk of cancer was higher in this population than in the general population. When treating patients who had undergone cholecystectomy, health care professionals need to be more aware of certain groups, such as patients aged under 65 years and those with certain cancers, such as colon cancer. In addition, attention should be paid to preventive follow-up that can effectively screen cancer occurrence. Further studies are needed on this topic.

## Figures and Tables

**Table 1:** Demographic characteristics of the cholecystectomy patients.

Characteristic	Cholecystectomy	
	N	%
**Total**	408,769	100
**Sex**		
Male	194,427	47.6
Female	214,842	52.4
**Age at operation (years)**		
20–29	20,992	5.1
30–39	66,365	16.2
40–49	85,647	21.0
50–59	92,652	22.7
60–69	71,866	17.6
70–79	53,747	13.1
≥80	17,500	4.3
Health insurance premium (KRW) *		
<40,000 (Q1–5)	67,069	16.4
40,000–70,000 (Q6–10)	73,798	18.1
70,000–120,000 (Q11–15)	99,234	24.3
>120,000 (Q16–20)	142,729	34.9
Missing	25,939	6.3
**Year of cholecystectomy**		
2008	33,557	8.2
2009	37,284	9.1
2010	40,067	9.8
2011	44,280	10.8
2012	47,292	11.6
2013	48,936	12.0
2014	49,406	12.1
2015	50,977	12.5
2016	56,970	13.9
**Total person-year**	2,002,230.34	

* The 20th percentile is based on the 2016 health insurance premium.

**Table 2:** Site-specific cancer incidence after cholecystectomy compared with that in the general population according to sex.

Cancer Site	ICD–10	Male					Female				
		Obs ^a^	Exp ^b^	Crude Rate ^c^	SIR	95% CI	Obs ^a^	Exp ^b^	CrudeRate ^c^	SIR	95% CI
**All cancers**	C00–C96	8483	7936.05	902.0	1.01	0.99–1.03	6572	6432.84	619.0	1.00	0.98–1.03
**Lip, oral cavity, pharynx**	C00–C14	132	147.79	14.04	0.83 *	0.69–0.97	42	51.89	3.96	0.79	0.55–1.03
**Stomach**	C16	1494	1464.25	158.9	0.95 *	0.90–1.00	560	659.18	52.74	0.83 *	0.76–0.90
**Colorecta**	C18–C20	1077	650.96	114.5	1.55 *	1.45–1.64	696	437.55	65.55	1.55 *	1.44–1.67
**Liver**	C22	1105	820.94	117.5	1.25 *	1.18–1.32	429	275.70	40.40	1.51 *	1.37–1.65
**Gallbladder and biliary tract**	C23–C24	293	228.91	31.15	1.22 *	1.08–1.36	196	194.47	18.46	1.01	0.87–1.15
**Other digestive organs**	C15, C17, C21, C25, C26	451	422.75	47.95	1.00	0.91–1.09	269	219.77	25.34	1.21 *	1.07–1.36
**Lung**	C33–C34	1174	1261.95	124.8	0.88 *	0.83–0.93	420	468.34	39.56	0.89 *	0.80–0.97
**Respiratory (other than C33–C34)**	C30–C39	117	115.82	12.44	0.94	0.77–1.11	23	25.66	2.17	0.87	0.51–1.23
**Bone and articular cartilage**	C40–C41	15	11.235	1.59	1.30	0.64–1.96	16	11.17	1.51	1.42	0.72–2.12
**Melanoma and skin**	C43–C44	202	146.41	21.48	1.33 *	1.15–1.51	245	181.08	23.08	1.40 *	1.23–1.58
**Mesothelial and soft tissue**	C45–C49	51	42.56	5.42	1.15	0.84–1.47	50	27.55	4.71	1.77 *	1.28–2.26
**Breast**	C50	4	5.30	0.43	0.72	0.01–1.43	1,127	985.92	106.1	1.12 *	1.06–1.19
**Female genital**	C51–C58	0	-	-	-	-	467	504.99	43.98	0.90 *	0.82–0.98
**Male genital**	C60, C62–C63	20	18.13	2.13	1.09	0.62–1.57	0	-	-	-	-
**Prostate**	C61	912	757.93	96.97	1.14 *	1.06–1.21	0	-	-	-	-
**Urinary tract**	C64–C68	553	473.41	58.80	1.11 *	1.02–1.20	159	158.19	14.98	0.98	0.83–1.14
**Eye, Brain and CNS**	C69–C72	77	53.97	8.19	1.37 *	1.07–1.68	91	48.52	8.57	1.83 *	1.46–2.21
**Thyroid**	C73	452	333.45	48.06	1.33 *	1.21–1.45	1,498	1540.71	141.1	0.95 *	0.90–1.00
**Other endocrine glands**	C74–C75	7	5.01	0.74	1.36	0.35–2.36	10	4.96	0.94	1.98	0.75–3.20
**Lymphoid and haematopoietic**	C81–C96	347	384.77	36.90	0.86 *	0.77–0.95	274	138.65	25.81	1.91 *	1.68–2.13

ICD: international classification of diseases, CNS: central nervous system, SIR: standardised incidence ratio * *p* < 0.05 ^a^ Observed numbers of cases ^b^ Expected numbers of cases ^c^ Crude rate per 100,000 person-years.

**Table 3:** Incidence rates of major cancers according to age in male patients who underwent cholecystectomy.

Male									
Age	20–34			35–64			≥65		
No.	Cancer Site	Crude Rate ^a^	SIR	Cancer Site	Crude Rate ^a^	SIR	Cancer Site	Crude Rate ^a^	SIR
	All cancers (C00–C96)	122.46	2.62 * (2.15–3.08)	All cancers (C00–C96)	607.74	1.36 * (1.32–1.40)	All cancers (C00–C96)	2083.93	0.90 * (0.87–0.92)
1	Thyroid (C73)	61.23	3.74 * (2.79–4.68)	Stomach (C16)	111.59	1.22 * (1.13–1.31)	Lung (C33–C34)	362.80	0.80 * (0.75–0.86)
2	Lymphoid and Haematopoietic (C81–C96)	10.21	1.28 (0.49–2.07)	Liver (C22)	99.23	1.57 * (1.45–1.69)	Stomach (C16)	360.07	0.89 * (0.83–0.95)
3	Colorectal (C18–20)	9.18	5.82 * (2.01–9.62)	Colorectal (C18–20)	76.75	2.16 * (1.97–2.35)	Prostate (C61)	284.05	0.98 (0.90–1.06)
4	Stomach (C16)	8.16	2.60 (0.80–4.41)	Lung (C33–C34)	60.05	1.39 * (1.25–1.53)	Colorectal (C18–20)	268.57	1.38 * (1.27–1.49)
5	Liver (C22)	6.12	3.96 (0.79–7.14)	Thyroid (C73)	54.59	1.31 * (1.17–1.45)	Liver (C22)	218.95	1.13 * (1.03–1.23)

SIR: standardised incidence ratio * *p* < 0.05 ^a^ Crude rate per 100,000 person-years.

**Table 4:** Incidence rates of major cancers according to age in female patients who underwent cholecystectomy.

Age	20–34 Years			35–64 Years			≥65 Years		
No.	Cancer Site	Crude Rate ^a^	SIR	Cancer Site	Crude Rate ^a^	SIR	Cancer Site	Crude Rate ^a^	SIR
	All cancers (C00–C96)	237.85	1.91 * (1.71–2.10)	All cancers (C00–C96)	577.35	1.07 * (1.03–1.10)	All cancers (C00–C96)	954.44	0.90 * (0.87–0.94)
1	Thyroid (C73)	128.41	1.75 * (1.51–1.99)	Thyroid (C73)	180.08	0.95 (0.89–1.00)	Colorectal (C18–20)	154.51	1.40 * (1.27–1.54)
2	Breast (C50)	34.79	2.48 * (1.82–3.13)	Breast (C50)	138.63	1.14 * (1.07–1.22)	Stomach (C16)	117.14	0.77 * (0.69–0.86)
3	Female genital (C51–C58)	32.89	2.48 * (1.81–3.16)	Female genital (C51–C58)	46.11	0.90 (0.80–1.00)	Liver (C22)	98.26	1.37 * (1.20–1.54)
4	Lymphoid and Haematopoietic (C81–C96)	14.55	3.94 * (2.33–5.55)	Colorectal (C18–20)	43.78	1.88 * (1.66–2.10)	Lung (C33–C34)	97.10	0.77 * (0.67–0.86)
5	Colorectal (C18–20)	8.22	5.71 * (2.61–8.81)	Stomach (C16)	38.35	0.96 (0.84–1.08)	Other digestive organs (C15, C17, C21, C25, C26)	70.13	1.11 (0.95–1.28)

SIR: standardised incidence ratio * *p* < 0.05 ^a^ Crude rate per 100,000 person-years.

## Data Availability

The data presented in this study are available on request to National Health Insurance Service database (https://nhiss.nhis.or.kr/bd/ab/bdaba031eng.do, accessed on 14 August 2018).
